# Is organizational intervention using Layered Voice Analysis effective in addressing operator mental health in call centers? A randomized controlled trial

**DOI:** 10.1093/joccuh/uiae047

**Published:** 2024-08-14

**Authors:** Naomichi Tani, Yoshihiro Takao, Sakihito Noro, Hiroaki Fujihara, Hisashi Eguchi, Kazuki Sakai, Takeshi Ebara

**Affiliations:** Department of Ergonomics, Institute of Industrial Ecological Sciences, University of Occupational and Environmental Health, Japan, 1-1 Iseigaoka, Yahatanishi-ku, Fukuoka, Kitakyushu 807-8555, Japan; ES Japan, Inc., 5F Dai-3 Kyoritsu Building, 2-50-9 Ikebukuro, Toshima-ku, Tokyo 171-0014, Japan; ES Japan, Inc., 5F Dai-3 Kyoritsu Building, 2-50-9 Ikebukuro, Toshima-ku, Tokyo 171-0014, Japan; Department of Ergonomics, Institute of Industrial Ecological Sciences, University of Occupational and Environmental Health, Japan, 1-1 Iseigaoka, Yahatanishi-ku, Fukuoka, Kitakyushu 807-8555, Japan; Department of Mental Health, Institute of Industrial Ecological Sciences, University of Occupational and Environmental Health, Japan, 1-1 Iseigaoka, Yahatanishi-ku, Fukuoka, Kitakyushu 807-8555, Japan; Department of Ergonomics, Institute of Industrial Ecological Sciences, University of Occupational and Environmental Health, Japan, 1-1 Iseigaoka, Yahatanishi-ku, Fukuoka, Kitakyushu 807-8555, Japan; Department of Ergonomics, Institute of Industrial Ecological Sciences, University of Occupational and Environmental Health, Japan, 1-1 Iseigaoka, Yahatanishi-ku, Fukuoka, Kitakyushu 807-8555, Japan

**Keywords:** digital health technology, mental health, organizational intervention, voice recognition, voice analysis, physiological signals

## Abstract

Objectives: To verify the effects of organizational interventions on mental health using Layered Voice Analysis (LVA).

Methods: A 12-week single-blind randomized controlled trial was conducted with call center operators. Sixty-six participants were randomly assigned to either a control group (*n* = 26), an LVA intervention group (*n* = 20), or a one-on-one intervention group (*n* = 20). The control group received general self-care information about preventing mental health problems from the Ministry of Health, Labour, and Welfare, Japan website. The organizational LVA intervention involved group sessions using participants’ voice calls with customers, whereas the one-on-one intervention consisted of meetings or consultations with participants and their supervisors to discuss preventing mental health issues at work. To verify the effectiveness of the intervention program, the Center for Epidemiologic Studies Depression Scale (CES-D) was administered 4 times (baseline, 4, 8, and 12 weeks) as the primary outcome, and the data were analyzed using a linear mixed model. The intervention of LVA was subdivided and analyzed into LVA ≥5 times and LVA ≤4 times out of the total 6 interventions.

Results: Compared with the control group, a significant CES-D reduction effect was observed at 8/12 weeks for the difference of coefficients (DOC; [β_int_ − β_ctrl_]) for the intervention of LVA ≥5 times (DOC −1.86 and −2.36, respectively). Similarly, even intervention LVA ≤4 times also showed a significant decrease of CES-D scores at 8/12 weeks (DOC −2.20 and −2.38, respectively).

Conclusions: An organizational intervention using LVA has the potential to reduce the risk of depression among call center operators.

## Teaser

**Key points**

1. **What is already known on this topic:** No previous studies have shown the effectiveness of organizational interventions in preventing workers’ mental health problems using voice emotion analysis.
2. **What this study adds:** In this study, a participatory intervention program using voice-emotion analysis technology was implemented to test the effectiveness of a mental health intervention for call center operators. The results showed that the participatory approach using voice analysis technology, Layered Voice Analysis, improved the mental health status of call center operators.
3. **How this study might affect research, practice, or policy:** Our study demonstrates the potential for using cutting-edge Layered Voice Analysis in mental health fields, helping to disseminate appropriate use of digital mental health technology.

## 1. Introduction

The World Health Organization (WHO) has reported that 15% of the world’s working-age population has a mental health disorder.^1^ In terms of disability-adjusted life years (DALYs), a WHO-defined assessment of healthy lives lost due to early death and disability,^2^ unipolar depressive disorder, one of the most common mental health disorders, is projected to become the leading DALY by 2030.^3^ Losses from mental health disorders alone amount to US$16.1 trillion, with a dramatic impact on productivity and quality of life.^4^ In other words, mental health disorders are an important issue in occupational health worldwide and are not irrelevant to Japan.

In a Special Survey on Industrial Safety and Health conducted by the Ministry of Health, Labour and Welfare, Japan, the percentage of workers who feel anxiety and stress in their current jobs and professional lives was quite high at 82.2%.^5^ In particular, interpersonal services, which involve emotional labor (Hochschild’s concept),^6^^,^^7^ are known to cause depression and burnout.^7^ According to Hochschild,^6^ some tertiary industries involve a type of work called “emotional labor,” which uses emotions to earn wages. This work requires people to assume desirable attitudes and facial expressions for customers and to consciously behave in ways that are appropriate for the situation. As a result, it is easy to become mentally stressed. Therefore, many workers in the service industry are absent from work for a month or more because of mental health problems.^5^ According to previous studies, among interpersonal services, call center operators are at an increased risk of depression due to exposure to malicious calls, civil complaints, verbal abuse, and sexual and provocative language from callers,^8^ with approximately 45.8% at risk of developing mental health disorders.^9^ Thus, operators working in call centers, who undertake emotional labor, represent an important occupational health issue.

As indicated in the guidelines based on a systematic review of mental health at work published by the WHO, not only line care and self-care but also organizational intervention approaches are key pillars for the prevention of workers’ mental health problems.^1^ Some studies and reviews have reported that organizational interventions that address psychosocial factors, including participatory approaches, have some effect on workers’ mental health, suggesting that they may contribute to reducing workers’ psychological stress and improving health outcomes,^10^^,^^11^ but the level of evidence in the WHO guidelines is “very low”^1^ and accumulation of high-quality social implementation evidence is required.

Recently, an increasing number of mental health intervention studies have used digital health technologies.^12^ For example, interventions with personal computers, tablets, smartphones, and email have been shown to promote mental health and reduce absenteeism in the workplace.^13^ Moreover, the effects of internet-based cognitive behavioral therapy and mindfulness have also been demonstrated in randomized controlled trials.^14^^‑^^16^ In addition, reports suggest that brief immersive virtual reality meditations, breathing exercises, and other forms of relaxation in the workplace may reduce stress.^17^ In Japan, however, occupational health research using digital health technologies has just begun. In Japan, digital health technology is defined as: “Digital health technology includes services that use technical algorithms (such as application software [apps], wearable devices, etc), and services that do not use technical algorithms (such as online counseling that solely relies on internet-based means)”^18^ and is categorized into 11 technical fields.^19^ Among these, fundamental research on voice emotion analysis has become active in recent years.^20^ Voice as a physiological signal has also received attention in recent years as an indicator of acute stress responses and has the potential to be applied as an upcoming digital mental health technology in occupational health. Although internet-based cognitive behavioral therapy has already been shown to be an effective mental health measure,^21^^,^^22^ new mental health measures using other digital health technologies are eagerly awaited. Particularly, there is no evidence for mental health interventions in the workplace that use voice emotion analysis. If this new mental health initiative using voice emotion analysis comes to fruition, it will have a significant impact on mental health care in the workplace.

Voice analysis technology, ie, Layered Voice Analysis (LVA), can measure and evaluate a speaker’s emotions and acute stress responses over time, making it possible to visualize his/her internal state. Developed by Nemesysco Ltd, LVA technology is an advanced process featuring complex algorithmic measurements that help in the detection of human emotional levels, and it is one of the most popular technologies used in deception detection, using voice fluctuations to detect psychological stress.^23^ The technology of LVA makes note of psychological distress levels, cognitive functions, and emotional reaction criteria for a deeper understanding of the mental and emotional state of an individual at any specific segment of time.^23^

Thus, we decided to devise a new organizational intervention program using LVA technology to prevent mental health problems produced by emotional labor under the high stress inherent in call centers and to test its effectiveness.

## 2. Methods

### 2.1. Study sample

Participants were recruited from the staff working at a call center in Kumamoto Prefecture, Japan. The study inclusion criteria were workers who worked in call centers (regular employees, contract employees, and hourly employees), were 18 to 65 years old, and had at least 3 months of service. The exclusion criteria were attending the hospital owing to mental illness, and managers or leaders at the baseline of the survey. We conducted the 12-week single-blind randomized controlled trial with call center operators. Details of the intervention program were explained to 240 employees, of whom 68 participated in the study and provided consent. A 3-arm program was used for the intervention program (ie, LVA intervention group, one-on-one interview group, and control group). Ultimately, 66 participants were randomly assigned to either the control group (*n* = 26), or an organizational intervention using LVA (*n* = 20), or a one-on-one meeting/consulting with their supervisor group (*n* = 20). Participants were asked to keep their assigned interventions unspoken (allocation concealment) and were also asked not to mention to others that they were participants in this study (performance bias). As no previous studies similar to the organizational intervention program used in this study were found, the sample size was calculated by substituting the effect size of a preventive mental health intervention study using an internet-based cognitive behavioral therapy application.^22^ Sample sizes were estimated based on the effect size of the Center for Epidemiologic Studies Depression Scale (CES-D)^24^^,^^25^ as quantitative variables = 0.796 (Hedges’ g), α = .05, and 1 − β = 0.8. We estimated that more than 57 participants would be required.

This study was approved by the Institutional Review Board of the University of Occupational and Environmental Health, Japan (ID23-001). All participants provided written informed consent at enrollment. This randomized controlled trial conformed to the CONSORT guidelines and was registered in the UMIN-CTR (ID: UMIN000051352).

### 2.2. Layered Voice Analysis specification

We used Emotional Signature Analysis Solution (ESAS), an LVA system provided by ES Japan, Inc. Previous studies have shown that psychological stress relates to voice pitch, pitch variability, and intensity.^26^ LVA, the basis of ESAS, analyzes speech waveforms and the degree of speech variability and is a system for determining the stress state of speakers.^23^^,^^27^ The LVA algorithm estimates each parameter’s values based on the speech waveforms in a conversation, using a unique formula to determine the percentage of waveforms characteristic of each emotional factor, the average time of appearance, and the deviation value. This information is Nemesysco’s trade secret and has not been disclosed to the public, but the technology has been registered as a patent (Patent Nos. US 6638217 and US 7165033). In other countries, it has already been introduced by police agencies to detect deceptive intent in their capability to solve crimes.^23^ In academic validation, the results of voice analysis using LVA revealed a correlation between emotional stress and the State–Trait Anxiety Inventory, a self-administered anxiety test, as well as a positive correlation between a parameter indicating the degree of psychological agitation and systolic blood pressure values, suggesting the possibility of assessing stress tolerance and psychological distress levels.^27^ The ESAS system measures human-generated speech once every 2 seconds (ie, 0.5 Hz) and determines 22 different emotional parameters such as content, sadness, aggression, stress, embarrassment, excitement, concentration, and energy by detecting minute tensions in speech waveform patterns. Thus, it is possible to visualize the stress or pleasure experienced by operators during conversations in any given scenario.

### 2.3. Main outcome

We assessed depressive symptoms with CES-D^24^ using a self-administered questionnaire. Participants were asked to respond to a self-administered questionnaire at 4 time points (baseline and at 4, 8, and 12 weeks). The CES-D consists of 20 items and assesses depression based on a total score. For each question item, respondents are asked to answer “no,” “1-2 days,” “3-4 days,” or “5 or more days” for the past week, and the degree of depression is rated by scores ranging from a maximum of 60 points to a minimum of 0 points.^24^^,^^25^ A score of 16 or higher on the CES-D indicates the presence of depressive symptoms.^24^^,^^25^ We used the difference in the CES-D scores between pre- and post-intervention as the outcome.

### 2.4. Procedures and intervention

**Figure 1:**
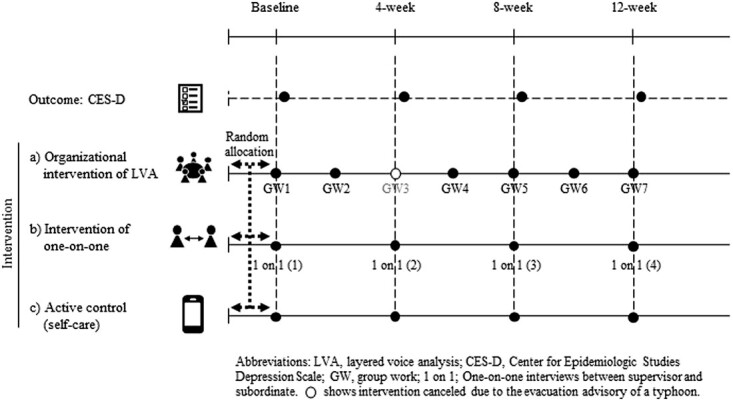
Scheme of intervention protocol for this study. GW3 was canceled because of the typhoon evacuation advisory. Thus, the total number of interventions was 6. We asked call center operators to complete a self-administered questionnaire (CES-D). The CES-D was answered after 4 points of intervention (baseline, 4, 8, and 12 weeks). CES-D, Center for Epidemiologic Studies Depression Scale.

As shown in Figure 1, a randomized controlled trial design was employed to assess the effect of an organizational intervention incorporating LVA feedback on the mental health of call center operators. At baseline, participants from each recruitment cluster were randomly assigned to either an organizational intervention using LVA, or an intervention of one-on-one meetings, or active control, and were asked to complete the CES-D. Group allocations were performed by study collaborators in a single-blinded manner (ie, concealed from participants). Participants were asked to complete the CES-D with a self-administered questionnaire 15 minutes before the end of the workday on 4 designated days (ie, baseline, 4, 8, and 12 weeks) during the 12-week study period.

The details of the interventions used in this study are shown in Table S1. Briefly, the LVA intervention group received a participatory intervention approach using LVA for 90 minutes per session every other week during the study period. The one-on-one intervention group received general line care as an individual interview by their supervisor, and the control group received general self-care information about preventing mental illness via the website of the Ministry of Health, Labour and Welfare, Japan. The URL for the information was delivered via email once a month.

Under the LVA intervention, the participation rate (adherence rate) was calculated by dividing the number of times the participants participated in group work by the number of times the intervention was implemented. The LVA protocol was scheduled for 7 interventions, but 1 was canceled owing to the typhoon evacuation advisory, resulting in a total of 6 interventions (Figure 1).

### 2.5. Statistical analysis

A linear mixed model analysis (LMM) was used to examine the effect on mental health of the organizational intervention using LVA. The analysis included 2 factors: condition of interventions (3 groups, comprising LVA, one-on-one, and active control) and time points of answering the CES-D (baseline and at 4, 8, and 12 weeks). Factors were treated as fixed effects in the model, and individuals were treated as random effects. We calculated each partial regression coefficient of the control group (β_ctrl_) and the intervention of LVA or one-on-one (β_int_), and if the differences of coefficients (β_int_ − β_ctrl_) were not equal to 0, then the result indicated a statistical intervention effect.^28^ Because this study aimed to evaluate the effectiveness of the organizational intervention program using a participatory approach with LVA, we applied an intention-to-treat analysis^29^ with initial allocation, even in the case of dropouts. To further clarify the effect of the intervention using LVA, those who participated in the LVA program intervention 2 or more times and those who did not were subdivided (ie, adherence rates of 66% or more or less) into LVA ≥5 times group and LVA ≤4 times group, respectively, for analysis. Missing data were processed using multiple imputations to minimize bias and maintain power.^30^ However, we excluded those with mental illness at baseline. This is because they were likely to have depressive symptoms (*n* = 2), which could have led to bias in the results of the analysis. Those who did not respond to the CES-D were excluded from the analysis (*n* = 1). All analyses were conducted using R version 4.2.2 (R Foundation for Statistical Computing, Vienna, Austria).

## 3. Results

**Figure 2:**
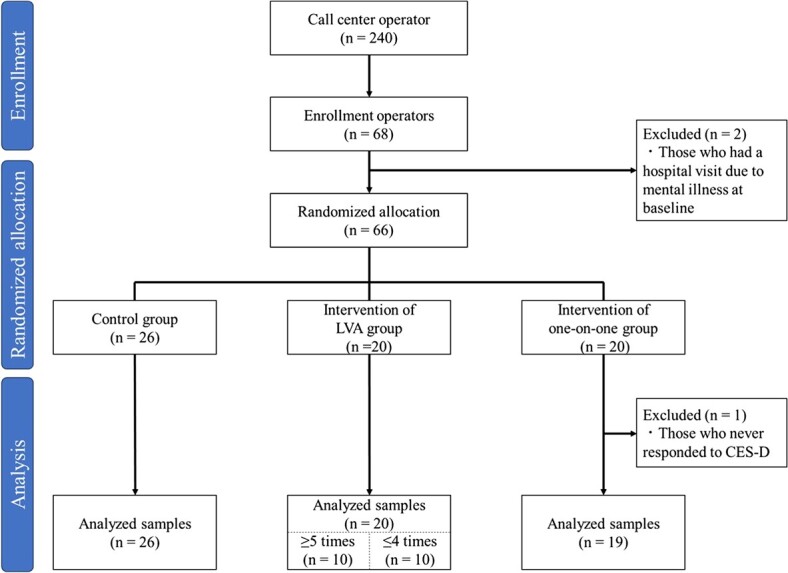
Participant flowchart.

Participants were recruited in June 2023 from the target companies, and 68 expressed their willingness to participate. A flowchart of the participants is shown in Figure 2. During follow-up, 5 participants (7.3%) became ineligible after leaving the organization, and 1 participant (1.4%) declined the consent form. Data from these participants were included in the analysis until retirement or withdrawal of consent. Additionally, those who visited hospitals due to mental illness at baseline (*n* = 2) and those who had never responded to the CES-D (*n* = 1) were excluded from the analysis. Sixty-five participants were included in the final analysis (Figure 2).

Participants’ characteristics are presented in Table 1. The mean (SD) age of the participants was 45.5 (14.7) years, the percentage of females was 88.7%, and the mean length of service was 2.7 years. Employment status was 49.2% full-time, 30.8% part-time, and 20% dispatched workers. No significant differences in these characteristics were observed between groups (Table 1).

**Table 1:** Characteristics of participants.

	**Control group**	**Intervention of LVA ≥5 times**	**Intervention of LVA ≤4 times**	**Intervention of one-on-one**	**Total**	***P* value**
	**(*n* = 26)**	**(*n* = 10)**	**(*n* = 10)**	**(*n* = 19)**	**(*n* = 65)**	
**Age,** ^**a**^ **y**	43.1 (14.3)	46.2 (13.1)	40.1 (16.6)	50.9 (14.4)	45.5 (14.7)	.225
**Sex: female** ^**b**^	21 (84.0)	10 (100.0)	9 (100.0)	15 (83.3)	55 (88.7)	.322
**Department** ^**b**^ ^**,**^ ^**c**^						.521
**Section 1**	12 (46.2)	4 (40.0)	4 (40.0)	12 (63.2)	32 (49.2)	
**Section 2**	14 (53.8)	6 (60.0)	6 (60.0)	7 (36.8)	33 (50.8)	
**Employment status** ^**b**^						.123
**Full-time**	10 (38.5)	6 (60.0)	8 (80.0)	8 (42.1)	32 (49.2)	
**Part-time**	11 (42.3)	1 (10.0)	0 (0.0)	8 (42.1)	20 (30.8)	
**Dispatched**	5 (19.2)	3 (30.0)	2 (20.0)	3 (15.8)	13 (20.0)	
**Years of experience** ^**a**^ **, y**	3.2 (2.3)	2.5 (2.2)	2.5 (1.7)	2.3 (1.9)	2.7 (2.1)	.483

Abbreviation: LVA, Layered Voice Analysis.

a Mean (SD), 1-way analysis of variance (ANOVA) test.

b *n* (%), χ^2^ test.

c The call center operators in Sections 1 and 2 have different procedures, because of the different companies they are contracted to.

The results of the LMM are presented in Table 2. Between the baseline and 4 weeks, significant inverse effects of the differences of coefficients (β_int_ − β_ctrl_) were found in the intervention of LVA ≥5 times (difference of coefficients, 1.36; 95% CI, 0.16-2.56) and intervention of one-on-one group (difference of coefficients, 2.55; 95% CI, 1.30-3.80). However, between the baseline and 8 weeks, significant intervention effects were observed, with the differences of coefficients in the intervention of LVA ≥5 times (difference of coefficients, −1.86; 95% CI, −3.50 to −0.22) and the intervention of LVA ≤4 times (difference of coefficients, −2.20; 95% CI, −3.74 to −0.66). Additionally, significant intervention effects were observed between baseline and 12 weeks, the differences of β_int_ – β_ctrl_ were decreased in the intervention of LVA ≥5 times (difference of coefficients, −2.36; 95% CI, −2.94 to −1.78), and the intervention of LVA ≤4 times (difference of coefficients, −2.38; 95% CI, −3.48 to −1.28). Furthermore, a significant decrease was observed in the intervention of a one-on-one group, showing a difference of coefficients (β_int_ − β_ctrl_) of −1.47 (95% CI, −2.41 to −0.53) between baseline and 12 weeks.

**Table 2:** Effect of an organizational intervention with LVA on mental health assessed by the CES-D between baseline and each point.

	**Estimated coefficient (β) (95% CI)**						**Difference of coefficients (β**_**int**_ **− β**_**ctrl**_**)**^**a**^					
	**4-weeks**		**8-weeks**		**12-weeks**		**4-weeks**		**8-weeks**		**12-weeks**	
**Control group (β**_**ctrl**_ **)**	1.34	(−1.89 to 4.57)	1.12	(−2.10 to 4.33)	1.80	(−1.38 to 4.99)						
**Intervention of LVA ≥5 times (β**_**int**_ **a)**	2.70	(−1.73 to 7.14)	−0.74	(−5.60 to 4.11)	−0.56	(−4.33 to 3.21)	**1.36**	**(0.16 to 2.56)**	**−1.86**	**(−3.50 to −0.22)**	**−2.36**	**(−2.94 to −1.78)**
**Intervention of LVA ≤4 times (β**_**int**_ **a')**	0.38	(−4.06 to 4.81)	−1.08	(−5.84 to 3.68)	−0.58	(−4.86 to 3.71)	−0.96	(−2.16 to 0.24)	**−2.20**	**(−3.74 to −0.66)**	**−2.38**	**(−3.48 to −1.28)**
**Intervention of one-on-one (β**_**int**_ **b)**	3.89	(−0.63 to 8.34)	0.75	(−4.14 to 5.65)	0.33	(−3.80 to 4.46)	**2.55**	**(1.30 to 3.80)**	−0.40	(−2.05 to 1.31)	**−1.47**	**(−2.41 to −0.53)**

Abbreviations: CES-D, Center for Epidemiologic Studies Depression Scale; LVA, Layered Voice Analysis. Estimated coefficients (β) are indicated as "β ctrl", "β int a", and "β int b" for the control, intervention of LVA, and intervention of one-to-one groups, respectively. However, the intervention of LVA group is shown as "β int a" for five or more interventions and "β int a' " for four or fewer interventions.

a Values in bold, *P* < .05.

## 4. Discussion

### 4.1. Interpretation

To our knowledge, this study presents the first findings on the impact of a participatory organizational intervention using LVA on the mental health of call center operators. The organizational intervention with LVA reduced overall CES-D scores, especially at 8/12 weeks. Moreover, there was a negligible difference in the intervention effect at 12 weeks between the intervention of LVA ≥5 times and the ≤4 times groups. The results indicate that even if busy call center operators are unable to participate in LVA intervention programs, if they can participate more than 60% of the time, mental health intervention effects can be achieved. Furthermore, the intervention of the one-on-one group showed a decrease in CES-D at 12 weeks, whereas the intervention of LVA showed a decrease at an earlier time (from 8 weeks). This indicates that participatory organizational intervention using LVA is more effective in preventing depressive symptoms at an early stage than one-on-one meetings with supervisors. However, the score of CES-D was significantly increased in both groups of LVA ≥5 times and one-on-one meetings at 4 weeks. This may have caused psychological stress owing to unfamiliarity with group work and interviews with supervisors. The fact that there was no significant increase at 4 weeks in the LVA ≤4 times group, which had less interpersonal contact, may support this hypothesis (Figure S1). Moreover, both the intervention of LVA ≥5 and one-on-one groups also showed a significant reduction in CES-D at 12 weeks. Therefore, even if there is a temporary increase in psychological stress due to interpersonal communication, both the LVA and supervisor interview interventions are expected to decrease mental stress.

WHO mental health guidelines suggested the organizational intervention effect as “very low,”^1^ and other studies have shown that organizational occupational health interventions have been ineffective in improving employee job satisfaction and fatigue.^31^ However, recent systematic reviews have shown that improving workers’ well-being requires staff participatory interventions that ensure workers’ voices are heard.^11^ Additionally, organizational work improvement with staff participation seems to improve not only the working environment but also the mental health of workers.^10^^,^^32^ This gap likely depends on the creation of a space in which staff can participate, speak voluntarily in the intervention program, and learn spontaneously. In other words, it is conceivable that it is important to set the conditions by which learning can take place. Particular attention has also been paid to intervention programs that help workers cope with stress.^33^

Organizational intervention was recently defined: “Organizational interventions are planned actions that primarily directly target working conditions with the aim of promoting and maintaining of the highest degree of physical, mental, and social well-being of workers in all occupations. In addition, organizational interventions are often primary and secondary prevention-focused, but may also include tertiary prevention.”^34^ In addition, as a precaution, “Interventions may be multimodal, including any combination of individual, manager, and organizational approaches such as workplace mental health promotion with an organizational component.”^34^ The participatory group discussion with LVA we provided was a program aimed at the primary prevention of mental illness among call center operators engaged in emotional labor. This intervention also aimed to promote occupational safety and health measures through top management initiatives. Although section leaders did not participate in the participatory group discussion, individual experiences were shared and discussed to improve personal skills on how to handle complaints, a stressor in call center operations. It was a useful intervention method that involved changing or improving an individual’s work process. Because workers who participated in the LVA intervention were involved in the steps, including action planning, implementation, evaluation, and review of their work skills,^34^ we believe that our approach was a participatory organizational intervention approach. In this context, LVA may have been effective in this intervention. In this study, LVA was used to visualize emotions and provide feedback in simulated exercises for various difficulties faced during operator work. Through the simulated exercise, operators could identify emotional feedback when their call work was handled well or not so well. This experience may have contributed to the development of skills for successfully handling stress during call work. Furthermore, through the participatory exchange of opinions in group work, it is possible that the tacit knowledge of coping with stress in call center work could have been converted into formal knowledge.

The primary strength of this study is that it is the first in Japan to investigate in a randomized controlled trial the impact of an organizational intervention using LVA on the mental health of call center operators. Owing to gaps in the evidence of the effectiveness of organizational interventions in occupational mental health, this study makes a significant contribution to occupational health research by providing new evidence. Another strength is that it verifies the effectiveness of an organizational intervention program using LVA technology, a type of voice emotion analysis that has been gaining attention in recent years.^20^ Incorporating a system of emotional feedback into workers’ organizational interventions would be useful as part of a new mental health prevention program.

### 4.2. Limitations and generalizability

The current study had some limitations. First, the small number of participants may have affected our results. The number of participants in all groups was lower than the sample size calculation, which may have resulted in a lack of power. Second, there is a potential mixed effect of intervention programs. Although this study was conducted to test the effectiveness of an organizational intervention, the improvement in individual stress-coping skills through LVA may also have influenced the results. Therefore, future studies are required to compare groups that use only LVA and focus solely on participatory organizational interventions. In addition, although this study was intended to confirm the effectiveness of the organizational LVA intervention applying intention-to-treat analysis, further research is needed to evaluate the efficacy of LVA itself compared with the differences between group sessions with and without LVA. Third, this study examined only short-term intervention effects. However, 12 weeks may have been insufficient to test the long-term effects of this intervention. Fourth, all the participants were adult employees and were biased toward a particular industry (selection bias). The generalizability of the results of this study should be considered in light of these points. Finally, although ESAS is based on the LVA system, it does not guarantee complete psychological stress detection accuracy. However, from a primary prevention perspective, even false positives do little harm to workers and may rather lead to enhanced mental health measures in the workplace.

## 5. Conclusions

An organizational intervention using LVA has the potential to reduce the risk of depression among call center operators. However, besides the small sample size of this study, the long-term effects have not been confirmed. Further research is required to resolve these issues.

## Supplementary Material

Web_Material_uiae047

## Data Availability

The data underlying this study will be shared with the corresponding author upon reasonable request.

## References

[1] World Health Organization (WHO) . Guidelines on mental health at work. Published September 2022. Accessed December 22, 2023. https://www.who.int/publications/i/item/9789240053052

[2] World Health Organization (WHO) . Disability-adjusted life years (DALYs). Published December 2020. Accessed December 22, 2023. https://www.who.int/data/gho/indicator-metadata-registry/imr-details/158

[3] World Health Organization (WHO) . The global burden of disease: 2004 update. Published March 2004. Accessed December 22, 2023. https://www.who.int/publications/i/item/9789241563710

[4] World Economic Forum . The global economic burden of non-communicable diseases. Published September 2011. Accessed December 22, 2023. https://www.weforum.org/publications/global-economic-burden-non-communicable-diseases/

[5] Ministry of Health, Labour and Welfare, Japan . Special survey on industrial safety and health. Published October 2023. Accessed December 22, 2023. https://www.mhlw.go.jp/toukei/list/r04-46-50b.html

[6] Williams A . Hochschild (2003) – the managed heart: the recognition of emotional labour in public service work. Nurse Educ Today. 2013;33(1):5-7. 10.1016/j.nedt.2012.07.006 22858305

[7] Eto S . Mental health at interpersonal service workplaces. J Rural Med. 2013;61(6):840-853 [in Japanese]. 10.2185/jjrm.61.840

[8] Kim YK , Cha NH. Correlations among occupational stress, fatigue, and depression in call center employees in Seoul. J Phys Ther Sci. 2015;27(10):3191-3194. 10.1589/jpts.27.3191 26644672 PMC4668163

[9] Toker MAS , Güler N. General mental state and quality of working life of call center employees. Arch Environ Occup Health. 2022;77(8):628-635. 10.1080/19338244.2021.1986462 34657581

[10] Tsutsumi A , Nagami M, Yoshikawa T, Kogi K, Kawakami N. Participatory intervention for workplace improvements on mental health and job performance among blue-collar workers: a cluster randomized controlled trial. J Occup Environ Med. 2009;51(5):554-563. 10.1097/JOM.0b013e3181a24d28 19365287

[11] Fox KE , Johnson ST, Berkman LF, et al. Organisational- and group-level workplace interventions and their effect on multiple domains of worker well-being: a systematic review. Work Stress. 2021;36(1):30-59. 10.1080/02678373.2021.1969476

[12] Lee K , Lee TC, Yefimova M, et al. Using digital phenotyping to understand health-related outcomes: a scoping review. Int J Med Inform. 2023;174:105061. 10.1016/j.ijmedinf.2023.105061 37030145

[13] Moe-Byrne T , Shepherd J, Merecz-Kot D, et al. Effectiveness of tailored digital health interventions for mental health at the workplace: a systematic review of randomised controlled trials. PLoS Digit Health. 2022;1(10):e0000123. 10.1371/journal.pdig.0000123 36812547 PMC9931277

[14] Imamura K , Furukawa TA, Matsuyama Y, et al. Differences in the effect of internet-based cognitive behavioral therapy for improving nonclinical depressive symptoms among workers by time preference: randomized controlled trial. J Med Internet Res. 2018;20(8):e10231. 10.2196/10231 30097419 PMC6109227

[15] Luangapichart P , Saisavoey N, Viravan N. Efficacy and feasibility of the minimal therapist-guided four-week online audio-based mindfulness program 'Mindful Senses' for burnout and stress reduction in medical personnel: a randomized controlled trial. Healthcare. 2022;10(12):2532. 10.3390/healthcare10122532 36554056 PMC9778772

[16] Lilly M , Calhoun R, Painter I, et al. Destress 9-1-1—an online mindfulness-based intervention in reducing stress among emergency medical dispatchers: a randomised controlled trial. Occup Environ Med. 2019;76(10):705-711. 10.1136/oemed-2018-105598 31138676

[17] Riches S , Taylor L, Jeyarajaguru P, Veling W, Valmaggia L. Virtual reality and immersive technologies to promote workplace wellbeing: a systematic review. J Ment Health. 2024;33(2):253-273. 10.1080/09638237.2023.2182428 36919828

[18] Eguchi H , Kojimahara N, Kanamori S, Imamura K, Tani N, Ebara T. The use of digital health technology to provide mental health services for employees in Japan. Environ Occup Health Practice. 2024;6(1):2023-0016-CT. 10.1539/eohp.2023-0016-CT PMC11020255 38258936

[19] Tani N , Fujihara H, Ishii K, et al. What digital health technology types are used in mental health prevention and intervention? Review of systematic reviews for systematization of technologies. J Occup Health. 2023;66(1):uiad003. 10.1093/joccuh/uiad003 PMC11020255 38258936

[20] Tani N , Yamaguchi C, Tsunemi M, et al. Ergonomic strategies for digital occupational health: preparing for the oncoming wave of technological innovation. Environ Occup Health Practice. 2024;6(1):2023-0028-CT. 10.1539/eohp.2023-0028-CT PMC11841802 40061365

[21] Reins JA , Buntrock C, Zimmermann J, et al. Efficacy and moderators of internet-based interventions in adults with subthreshold depression: an individual participant data meta-analysis of randomized controlled trials. Psychother Psychosom. 2021;90(2):94-106. 10.1159/000507819 32544912

[22] Xiong J , Wen JL, Pei GS, Han X, He DQ. Effectiveness of internet-based cognitive behavioural therapy for employees with depression: a systematic review and meta-analysis. Int J Occup Saf Ergon. 2023;29(1):268-281. 10.1080/10803548.2022.2043647 35172706

[23] Maniar K , Rathod S, Kumar A, Jain SK. A forensic psychological study for detection of deception in financial fraud calls on layered voice analysis (LVATm). Int J Indian Psychol. 2022;10(1):572-585. 10.25215/1001.057

[24] Radloff LS . The CES-D scale: a self-report depression scale for research in the general population. Appl Psychol Meas. 1977;1(3):385-401. 10.1177/014662167700100306

[25] Shima S , Shikano T, Kitamura T, Asai K. New self-rating scales for depression [in Japanese]. Seishin-igaku. 1985;27(6):717-723

[26] Hollien H . Vocal indicators of psychological stress. Ann N Y Acad Sci. 1980;347(1 Forensic Psyc):47-72. 10.1111/j.1749-6632.1980.tb21255.x 6930921

[27] Nemoto K , Tachikawa K, Tanimukai S, et al. Detection of psychological stress using layered voice analysis technology [in Japanese]. Seishin Igaku. 2008;50(7):959-967

[28] Yamamoto K , Ebara T, Matsuda F, et al. Can self-monitoring mobile health apps reduce sedentary behavior? A randomized controlled trial. J Occup Health. 2020;62(1):e12159. 10.1002/1348-9585.12159 32845553 PMC7448798

[29] Moher D , Hopewell S, Schulz KF, et al. CONSORT 2010 explanation and elaboration: updated guidelines for reporting parallel group randomised trials. BMJ. 2010;340:c869. 10.1136/bmj.c869 20332511 PMC2844943

[30] White IR , Royston P, Wood AM. Multiple imputation using chained equations: issues and guidance for practice. Stat Med. 2011;30(4):377-399. 10.1002/sim.4067 21225900

[31] Framke E , Sørensen OH, Pedersen J, Rugulies R. Effect of a participatory organizational-level occupational health intervention on job satisfaction, exhaustion and sleep disturbances: results of a cluster randomized controlled trial. BMC Public Health. 2016;16(1):1210. 10.1186/s12889-016-3871-6 27899101 PMC5129592

[32] Bakhuys Roozeboom MC , Schelvis RMC, Houtman ILD, Wiezer NM, Bongers PM. Decreasing employees' work stress by a participatory, organizational level work stress prevention approach: a multiple-case study in primary education. BMC Public Health. 2020;20(1):676. 10.1186/s12889-020-08698-2 32404084 PMC7218833

[33] Kaveh MH , Mehrazin F, Cousins R, Mokarami H. Effectiveness of a transactional model-based education programme for enhancing stress-coping skills in industrial workers: a randomized controlled trial. Sci Rep. 2023;13(1):5076. 10.1038/s41598-023-32230-2 36977726 PMC10050170

[34] Sakuraya A , Iida M, Imamura K, et al. A proposed definition of participatory organizational interventions. J Occup Health. 2023;65(1):e12386. 10.1002/1348-9585.12386 36737041 PMC9897955

