# Supplementary material for: Is organizational intervention using Layered Voice Analysis effective in addressing operator mental health in call centers? A randomized controlled trial
Source: J Occup Health. 2024 Aug 14;66(1):uiae047. doi: 10.1093/joccuh/uiae047 (PMC11460070; doi:10.1093/joccuh/uiae047)
Supplement: Web_Material_uiae047 [file web_material_uiae047.zip › Supp_Table1.docx]

| **Supplementary Table 1. Details of the interventions in this study** | |
| --- | --- |
| Groups | Intervention |
| Intervention of LVA | The organizational intervention of Layered Voice Analysis (LVA) received group work for 90 minutes per session every other week during the study period (i.e., seven times). The first two interventions were designed as icebreaker sessions to reduce tension among participants, and the subsequent five interventions were group work programs using LVA of their voice calls with customers. In this group work, the participants looked back on using visualized data of their internal changes of emotions analyzed by LVA during talks with customers. The voice call data used was requested to be selected by themselves focusing on good practices of flexible responses according to circumstances. Reviewing and sharing their experience within the group by using LVA technology with each other is intended to convert tacit knowledge of coping against communication into formal knowledge each operator has. In addition, a SNS-based mutual encouragement mechanism was established.   1. GW1: Icebreaker session. Self-introduction, and orientation using LVA were performed. Each group experienced a simple trial training of calls with simulated customers using LVA. 2. GW2: They used LVA to analyze the audio of their self-introductions and were group discussions. Through this experience, they received visualized feedback on their generated emotion data from their voices. 3. GW3: The event was canceled due to the evacuation advisory of a typhoon. 4. GW4-7: They had role-play lessons with simulated scenarios using LVA and shared some examples that their voice recording of calls with customers analyzed. |
| Intervention of one-on-one | This group received general one-on-one meetings/consulting with call center operators and their supervisors to have conversations about preventing mental health issues at work. These interventions were conducted once a month (i.e., four times during the study period). |
| Control | The control group received general self-care information about preventing mental health via the website of the Ministry of Health, Labour and Welfare, Japan. The URL of the information was delivered by e-mail once a month (i.e., four times), and they were requested to do self-care based on the guidance themselves. |
