# Supplementary figures and images for: Is organizational intervention using Layered Voice Analysis effective in addressing operator mental health in call centers? A randomized controlled trial
Source: J Occup Health. 2024 Aug 14;66(1):uiae047. doi: 10.1093/joccuh/uiae047 (PMC11460070; doi:10.1093/joccuh/uiae047)

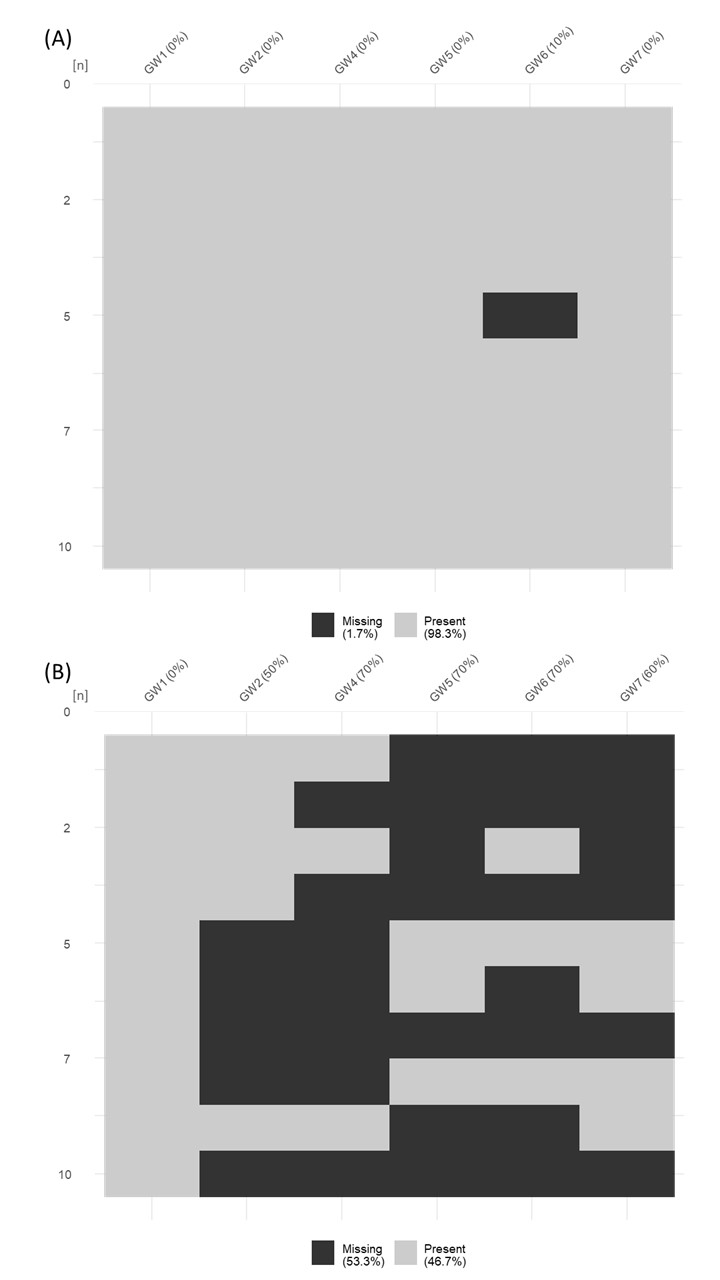

Supplement: Web_Material_uiae047 [file web_material_uiae047.zip › Supplementary_Figure1.jpg]
